# Searching for sliding surfaces in multi-level loess slopes based on the brachistochrone

**DOI:** 10.1038/s41598-023-33559-4

**Published:** 2023-04-20

**Authors:** Kui-bin Yang, Yan-peng Zhu

**Affiliations:** 1 School of Civil Engineering and Architecture, Shaanxi University of Technology, Hanzhong, 723001 Shaanxi China; 2 Key Laboratory of Disaster Mitigation in Civil Engineering of Gansu Province, Lanzhou University of Technology, Lanzhou, 730050 China; 3 Western Engineer Research Center of Ministry of Education for Disaster Mitigation in Civil Engineering, Ministry of Education, Lanzhou University of Technology, Lanzhou, 730050 China

**Keywords:** Civil engineering, Applied mathematics

## Abstract

As a key problem in slope-stability analysis, searching for potential sliding surfaces has attracted the attention of experts and scholars for a long time. However, the commonly used sliding surface curves are only considered in terms of shape approximation and lack physical significance. The search process involved in stability analysis of multi-level slopes is complex and a large amount of calculation is required. In order to solve this problem, this paper proposes a new sliding surface form based on physical interpretation of the brachistochrone, and establishes a search model for the brachistochrone sliding surface of a multi-level loess slope. At the same time, in order to further expand the search range and find a more ideal potential sliding surface curve shape and position with a lower safety factor, we recommend continuing the sliding-surface search after the brachistochrone is improved. We compared the calculation results for the position of the potential sliding surface and the stability safety factor with the corresponding results for an arc sliding surface (in combination with a calculation example) to verify its rationality. The approach offered here not only provides a new choice of sliding surface curve form for slope-stability analysis, but also significantly improves search efficiency for potential sliding surfaces of multi-level loess slopes.

## Introduction

As a classical problem in soil mechanics, slope-stability analysis is an ancient and dynamic subject in the field of geotechnical engineering^1,2^. The key to this type of analysis is finding the best method to evaluate stability, which involves two key problems: calculation of the stability safety factor and searching for potential sliding surfaces^3,4^. The search for potential sliding surfaces is the main purpose of slope-stability analysis. Once a potential sliding surface has been determined, the safety of the slope can be determined according to the shear strength of the potential sliding surface; therefore this determination has always been the focus of experts and scholars in the field of slope stability^5–8^. At the same time, with the continuous growth of the construction scale of transportation infrastructure, a large number of high and steep slopes are appearing. The corresponding instability accidents occur repeatedly^9^. In order to ensure slope safety, the actual engineering design is often based on comprehensive considerations of safety and economy. High and steep slopes, or those with poor stability, are graded to form a stepped multi-level slope. This approach is generally utilized in loess areas, especially along roadsides^10^. The failure mode of multi-stage slopes is different from that of single-stage slopes. The potential sliding surface may be on a certain secondary slope or run through the whole slope^11,12^. Therefore, finding an accurate and rapid way to search for the potential sliding surface is very relevant.

In research on potential sliding surfaces, experts and scholars have achieved many fruitful results. Among many methods, the circle center grid search method based on an arc is the one most favored by the engineering community, and the related research is also the most abundant^13–15^. At the same time, as our understanding of slopes improves, we have gradually realized that the most dangerous type of sliding surface is not an arc, but rather other regular or irregular curve forms similar to the arc. Therefore, many scientists are looking for other sliding surface forms that can replace the circular arc sliding surface model; the logarithmic helix is the most recognized form^16–18^. These two regular sliding surface curve forms have been verified as safe and feasible in many engineering scenarios, and have accumulated a lot of exposure in theory and practice, but there are also some deficiencies in this approach. For one thing, these forms are only approximations of the actual shape, and lack physical specificity. Also, the position of the rotation center of the sliding surface and the distance between the rotation center and the curve need to be determined during the search process. The search time will increase as search range and search accuracy grow. Due to their multiple levels, the position of potential sliding surfaces in multistage slopes is very uncertain, the search range is larger, and the search workload increases significantly. At the same time, combining a potential sliding surface search with intelligent optimization algorithms enables searches for arbitrary sliding surfaces; examples of these algorithms include genetic algorithms, ant-colony algorithms, and whale-optimization algorithms^19–23^. Obviously, intelligent algorithms require corresponding analysis software for application, and the calculation process is more complex when they are used. On the whole, the existing potential-sliding-surface search methods are very rich, and various optimization methods have developed rapidly, but their application in the field of practical engineering is still quite limited^24^.

Based on the above analysis, it is evident that determination of potential sliding surfaces is not only affected by the calculation of the safety factor, but also related to the particular sliding surface and search strategy. The focus of the existing research is too concentrated, research on the topic is scant, and there is a lack of exploration of other possible sliding surface forms. Choosing a curve form only involves approximating the shape, and does not take into consideration whether the curve has actual physical meaning. Therefore, it is necessary to explore further and find a sliding surface form with physical meaning and a more efficient search method. In this paper, a new sliding surface search model for multi-level loess slopes is established based on the brachistochrone, and its rationality is verified by an example calculation.

## The background of the brachistochrone sliding surface

**Figure 1:**
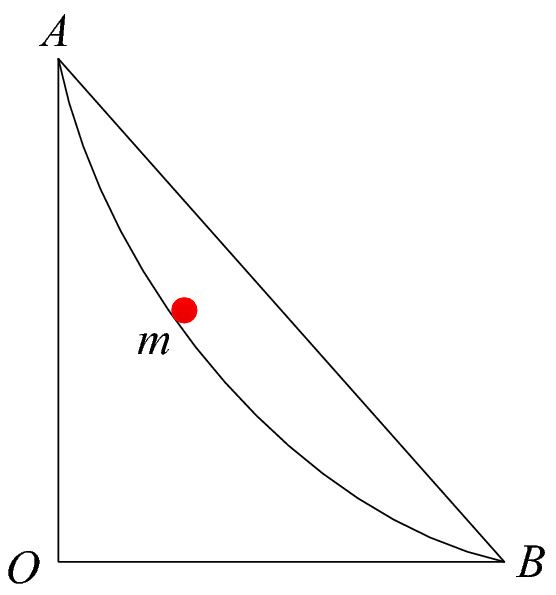
Schematic diagram of the brachistochrone.

The Italian scientist Galileo first raised the problem of the brachistochrone in 1630, and came to the wrong conclusion that the curve is a circular arc. Subsequently, the European mathematics community was involved in intense discussions on this question. Many mathematicians such as John and Jacob Bernoulli, Newton, Leibniz, and L'Hôpital reached a common correct conclusion, namely that the brachistochrone is not a straight line or an arc, but a cycloid. It is the optimal path by which a point mass goes from one point to another point that is not vertically below the starting point. It gives the fastest energy conversion and the shortest conversion time, as shown in Fig. 1.

It can be seen from Fig. 1 that the brachistochrone not only has a specific physical meaning, but also requires only two coordinates to determine the curve. If it can be used as the slope sliding surface curve form, this would inevitably reduce computational workload and improve search efficiency.

## Model for searching for the brachistochrone sliding surface

The failure mode of a multi-level slope is different from that of a single-grade slope. The sliding surface may occur on a certain secondary slope or run through the whole slope. Therefore, the search scope for the potential sliding surface of a multi-level slope is larger, and so is the uncertainty of sliding surface location. The safety factor and potential sliding surface of each step deserve attention^25^.

### Two assumptions

1. Many scholars directly assume the sliding surface to be an arc or logarithmic spiral in their studies^26–31^. They do this mainly because circular arcs and logarithmic spirals are similar in shape to the sliding surface of the slope. In order to establish the brachistochrone sliding surface search model for multi-level loess slopes, we can simply directly assume the form of the sliding surface to be the brachistochrone.
2. The potential sliding surface of a clay slope passes through the toe of the slope, so we will follow other scholars in assuming this to be the case^32–34^.

### Establishment of the potential sliding surface search model.

**Figure 2:**
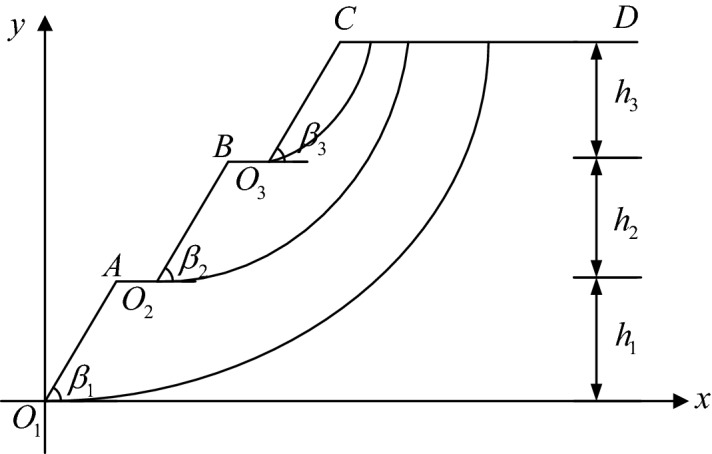
Schematic diagram of the search model for the sliding surface of a multi-level loess slope.

As shown in Fig. 2, the sliding surface search model is introduced with a three-level slope as an example. $O_{1}$,$O_{2}$, and $O_{3}$ are the slope toes of each step, and the origin of the plane rectangular coordinate system is at the bottom slope toe *O*_1_.

Given that the stability of each step of the multi-level slope is important, in order to search for potential sliding surfaces of slopes at all levels, the toe of each step is used as the sliding-out point, and the sliding-in point position is moved along the slope surface. For example, for the second step, we take *O*_2_ as the slip-out point; the slip-in point moves along $O_{2} B$,$BO_{3}$,$O_{3} C$, and CD. In this search process, the sliding surface corresponding to the minimum safety factor is taken as the potential sliding surface of the slope.

**Figure 3:**
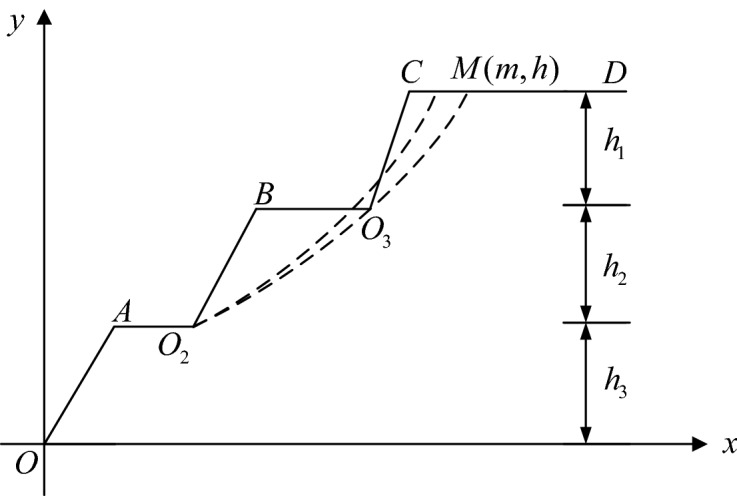
Schematic diagram of broken arc.
  Let us take the search for the potential sliding surface of the second-level slope pictured in Fig. 3 as an example; point *O*_2_ is the slip-out point of the potential sliding surface of the slope at this level. When the sliding surface happens to pass through point $O_{3}$, let its vertex coordinate be (*m*, *h*), where $h$ is the known value and $m$ is the unknown. Clearly, substituting the coordinates of point *O*_3_ and point $M$ into formula (1) can obtain the value of *m*, and then determine the position of point *M*.
  In order to avoid the occurrence of the broken arc phenomenon, the movement range of the curve vertex of the sliding surface should be controlled during the search process; that is, the search interval $O_{3} C$ and $CM$ should be excluded, and the curve vertex of the sliding surface should only move along *O*_2_*B*, *BO*_3_, and $MD$.

1. Sliding surface parameter equation
  In the coordinate system shown in Fig. 2, we assume that the coordinate of the slip-out point of the brachistochrone sliding surface is $\left( {a,b} \right)$ and the coordinate of the sliding-in point is $\left( {m,h} \right)$. Then the parameter equation of the brachistochrone sliding surface is as follows:
  1 $$\left\{ \begin{gathered} x = r(t - \sin t) + m \hfill \\ y = - r(1 - \cos t) + h \hfill \\ \end{gathered} \right.$$
  Obviously, $r$ and the value range of independent variable $t$ should be obtained to determine the parametric equation of the brachistochrone sliding surface. The solution process is as follows.
  ① Substituting the sliding-out point coordinate (*a*, *b*) into the sliding surface parameter equation gives the boundary value $t^{^{\prime}}$ of the independent variable at the sliding-out point; the value range of the independent variable *t* is $\left( {t^{^{\prime}} ,0} \right)$.
  ② Then, substituting the coordinate (*a*, *b*) of the sliding-out point and the boundary value *t* of the independent variable at the sliding-out point into the parametric equation, the value of *r* can be obtained.
    From the parameter equation of the brachistochrone sliding surface, it can be seen that in the process of moving the apex position (*m*, *h*) of the sliding surface curve along the slope line to find the potential sliding surface of each level of slope, the brachistochrone only requires an unknown quantity of the abscissa $m$ of the vertex of the sliding surface. However, it is clear from the curve equations of the circular arc and logarithmic spiral that when the potential sliding surface of each step of the slope is assumed to pass through the slope toe of the step, determination of the circular arc and logarithmic spiral requires two unknowns. Therefore, using the brachistochrone for the sliding surface will significantly reduce the search workload and improve search efficiency.

(B) Exclusion of "broken arc"
  It is not difficult to see from the diagram of the multi-level loess slope sliding surface search model that when the vertex is moved, the sliding surface curve will intersect with the slope line, as shown in Fig. 3. This phenomenon is called "broken arc". Obviously, the "broken arc" sliding surface does not exist, and the calculated safety factor does not have any significance, so it should be excluded from the sliding surface search process.

### Safety factor solution

The Morgenstern-Price method, Janbu method, and Sarma method are common approaches to finding the stability safety factor of non-circular arc sliding surfaces. In this paper, we use the simplified Janbu method to calculate the safety factor. The specific calculation formula is as follows:

2 $$F_{s} = \frac{{\sum\limits_{i}^{n} {(c_{i} l_{i} \cos \theta_{i} + W_{i} \tan \varphi_{i} )\frac{1}{{m_{\theta i} }}} }}{{\sum\limits_{i}^{n} {W_{i} \sin \theta_{i} } }}$$

3 $$m_{\theta i} = \cos \theta_{i} + \frac{{\sin \theta_{i} \tan \varphi_{i} }}{{F_{s} }}$$

where $c_{i}$ is the cohesive force of soil; $\varphi_{i}$ is the internal friction angle of soil; $W_{i}$ is the weight of the soil strip; $l_{i}$ is the curve length at the bottom of the soil strip; and $\theta_{i}$ is the angle between the tangent of the sliding surface at the center point of the bottom of the soil strip and the horizontal plane.

**Figure. 4:**
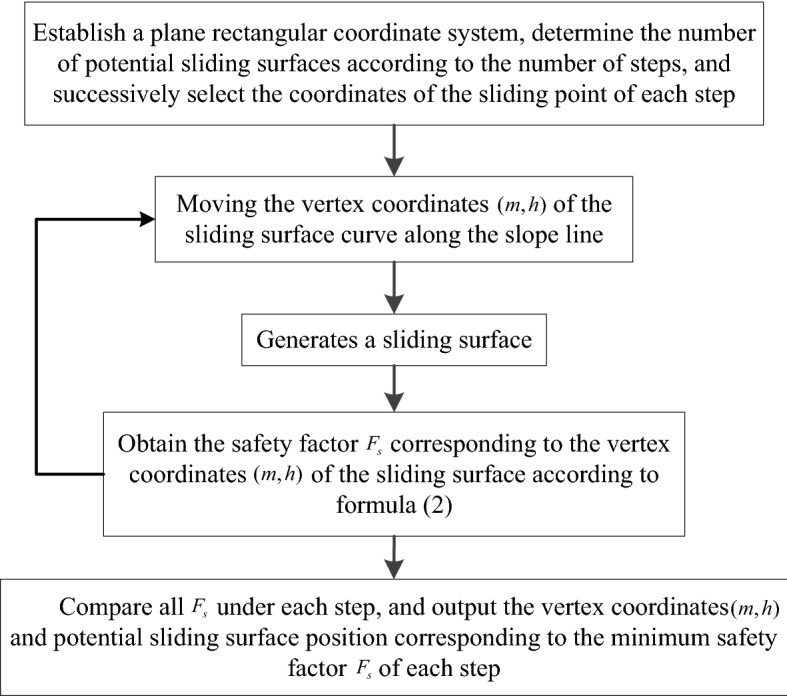
Flowchart of sliding surface search.

1. The angle between the tangent of any point on the sliding surface and the horizontal plane
  For the brachistochrone sliding surface, the derivative at any point on the sliding surface curve can be obtained through its parametric equation as:
  4 $$\frac{dy}{{dx}} = \frac{dy/dt}{{dx/dt}} = \frac{ - r\sin t}{{r - r\cos t}} = \tan (90^\circ + \frac{t}{2})$$
  Therefore, the angle between the tangent at any point of the sliding surface and the horizontal plane is:
  5 $$\theta_{i} = \frac{{(\pi + t_{i} )}}{2}$$

(B) Weight of soil strip
  In a multi-level slope, the slope-surface line can be represented by a piecewise function, so the gravity of the $i$th soil strip can be calculated as follows:
  6 $$W_{i} = \gamma_{i} (S_{1i} - S_{2i} )$$
  7 $$S_{1i} = \left\{ \begin{gathered} \int_{{x_{i - 1} }}^{{x_{i} }} {\frac{x}{{n_{1} }}dx \quad \, (0 \le x \le n_{1} h_{1} )} \hfill \\ \int_{{x_{i - 1} }}^{{x_{i} }} {h_{1} } dx \, \quad (n_{1} h_{1} \le x \le n_{1} h_{1} + s_{1} ) \hfill \\ \int_{{x_{i - 1} }}^{{x_{i} }} {\left[ {\frac{1}{{n_{2} }}(x - n_{1} h_{1} - s_{1} ) + h_{1} } \right]dx \, \quad (n_{1} h_{1} + s_{1} \le x \le n_{1} h_{1} + s_{1} + n_{2} h_{2} )} \hfill \\ \int_{{x_{i - 1} }}^{{x_{i} }} {h_{2} } dx \, \quad (n_{1} h_{1} + s_{1} + n_{2} h_{2} \le x \le n_{1} h_{1} + s_{1} + n_{2} h_{2} + s_{2} ) \hfill \\ ... \, ...... \hfill \\ \int_{{x_{i - 1} }}^{{x_{i} }} {h_{n} } dx \quad \, (n_{1} h_{1} + s_{1} + n_{2} h_{2} + s_{2} + ... + n_{n} h_{n} \le x) \hfill \\ \end{gathered} \right.$$
  8 $$S_{2i} = \int_{{t_{i - 1} }}^{{t_{i} }} {\left\{ {[ - r(1 - \cos t) + h](r - r\cos t)} \right\}} dt$$
  where $\gamma_{i}$ is the weight of the ith soil strip; $S_{1i}$ is the area enclosed by the slope line corresponding to soil strip $i$ and the $x$-axis of the coordinate system; $S_{2i}$ is the area enclosed by the brachistochrone sliding surface curve corresponding to soil strip $i$ and the *x*-axis of the coordinate system; $n_{1}$, $n_{2}$…$n_{n}$ are the slope rates of slopes 1, 2…$n$; $h_{1}$, $h_{2}$…$h_{n}$ are the slope heights of slopes 1, 2…*n*; $s_{1}$, $s_{2}$…$s_{n}$ are the platform widths of slopes 1, 2…*n*; $x_{i - 1}$, $x_{i}$ are the *x*-axis abscissa corresponding to the left and right boundaries of soil strip $i$; and $t_{i - 1}$, $t_{i}$ are the value of the independent variable *t* of the brachistochrone corresponding to the left and right boundaries of soil strip $i$.
(C) Curve length at the bottom of the soil strip
  According to the arc-length calculation method in the parametric equation, the curve length at the bottom of soil strip $i$ can be obtained as follows:
  9 $$l_{i} = \int_{{t_{i - 1} }}^{{t_{i} }} {\sqrt {[r(1 - \cos t)]^{2} + (r\sin t)^{2} } } dt$$
(D) Calculation-program implementation
  All the unknown quantities required to solve for the safety factor can be determined using the above formula, so we compiled the potential sliding surface search program with MATLAB. The program searched for the sliding surface by moving the *M* point position along the slope line, and finally found the coordinates of the curve vertex corresponding to the minimum safety factor, drawing the potential sliding surface curve. The specific process is shown in Fig. 4.

## Searching the improved brachistochrone sliding surface

### Defining the improvement factor

In order to further expand the search range, one must find a shape and position for the potential sliding surface curve that is more ideal and has a smaller safety factor. We introduce the parameter $\mu$ into the original equation and define it as an improvement coefficient. The classical brachistochrone is thus improved into the following formula:

10 $$\left\{ \begin{gathered} x = r(t - \sin t) + m \hfill \\ y = - \mu r(1 - \cos t) + h \hfill \\ \end{gathered} \right.$$

It can be seen from the improved sliding surface curve equation that the sliding surface changes with the value of *μ*, and the curve corresponding to the minimum safety factor is the potential sliding surface to be searched.

### Physical meaning of the improvement coefficient

**Figure 5:**
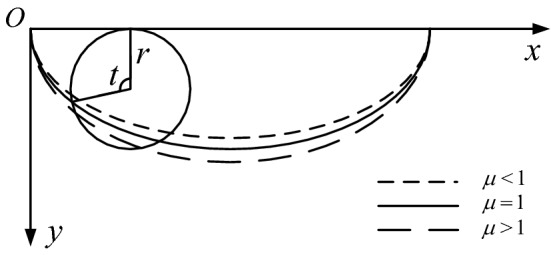
Schematic diagram of cycloid.

The curve of the brachistochrone is actually the trajectory formed by a certain point on a particular circle when the circle advances along a fixed straight line (a typical cycloid), as shown in Fig. 5. From the formation of the cycloid, it can be seen that the improvement coefficient *μ* has the following physical significance:

1. When *μ * < 1 and the circle advances along a fixed straight line, it is accompanied by sliding in the direction of advancement, which accelerates the circle’s rolling along the fixed straight line.
2. When *μ*  = 1, the curve is the classic brachistochrone;
3. When *μ*  > 1 and the circle advances along a fixed straight line, it is accompanied by a sliding movement opposite to the direction of advancement, which delays the circle’s rolling along the fixed straight line.

### Range of the improvement coefficient

It can be seen from the improved curve equation that when $t = 0$, the curve passes through point (*m*, *h*), but it does not always pass through $\left( {0,0} \right)$ with all possible values of *μ*. Therefore, it is necessary to discuss the value range of *μ*. The value can be obtained by substituting point (0, 0) into formula (10).

11 $$\mu = \frac{h(t - \sin t)}{{m(\cos t - 1)}}$$

Clearly, the value range of *μ* depends on the position of the sliding surface slip-in point (*m*, *h*). Based on formula (10), the specific interval of the improvement coefficient can be written as follows:

12 $$0 < \mu \le \frac{\pi h}{{2m}}$$

### Calculating the safety factor

As the curve equation of a sliding surface changes, the unknown parameters related to the safety factor in formula (2) will also change, so these parameters need to be determined separately.

**Figure. 6:**
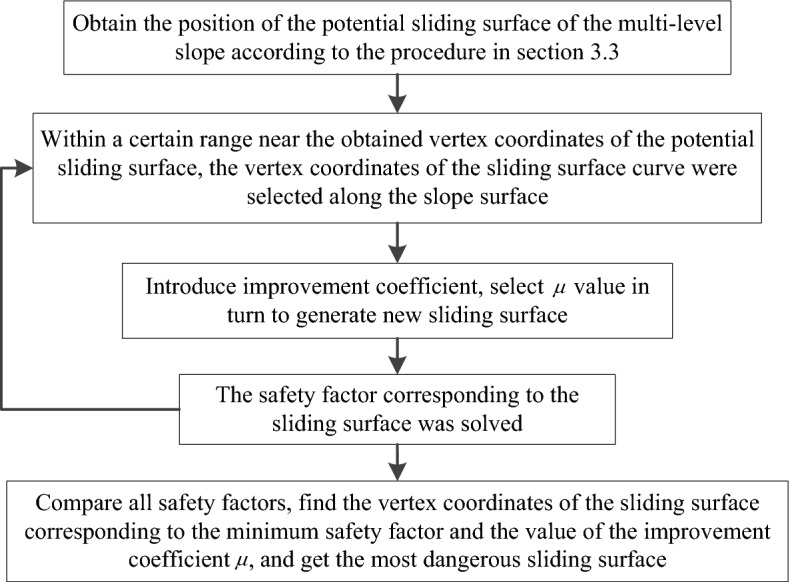
Flowchart of the improved sliding surface search.

(1) The angle between the tangent of any point on the sliding surface and the horizontal plane.
  From the improved brachistochrone parametric equation, the derivative at any point on the sliding surface curve can be calculated as follows:
  13 $$\frac{dy}{{dx}} = \frac{dy/dt}{{dx/dt}} = \frac{ - \mu r\sin t}{{r - r\cos t}} = \frac{ - \mu \sin t}{{1 - \cos t}}$$
  Therefore, the angle between the tangent line at any point on the sliding surface and the horizontal plane can be calculated by:
  14 $$\theta_{i} = \arctan \frac{{ - \mu \sin t_{i} }}{{1 - \cos t_{i} }}$$
Soil-strip weight
  In calculation formula (6) for soil-strip weight, only $S_{2i}$ changes, and the area enclosed by the sliding surface curve of the improved brachistochrone and the *x*-axis of the coordinate system is calculated as follows:
  15 $$S_{2i} = \int_{{t_{i - 1} }}^{{t_{i} }} {\left\{ {[ - r\mu (1 - \cos t) + h](r - r\cos t)} \right\}} dt$$
Curve length at the bottom of the soil strip
  Based on the parametric equation for finding arc length, the curve length at the bottom of soil strip $i$ corresponding to the improved brachistochrone can be calculated by:
  16 $$l_{i} = \int_{{t_{i - 1} }}^{{t_{i} }} {\sqrt {(r - r\cos t)^{2} + (\mu r\sin t)^{2} } } dt$$
Calculation program implementation
  Through a global search for the potential sliding surface, as described in section "Model for searching for the brachistochrone sliding surface", the positions of the potential sliding surface corresponding to each step in the multi-level slope can be roughly determined. On this basis, and using the obtained sliding surface vertex coordinates to define the central point, the sliding surface vertices are selected successively along the slope lines on each side at a certain search interval, and the improvement coefficient is introduced into the sliding surface parameter equation corresponding to each sliding point to continue the search for the potential sliding surface. The specific calculation process is shown in Fig. 6.

## Example analysis

### Example overview

**Figure 7:**
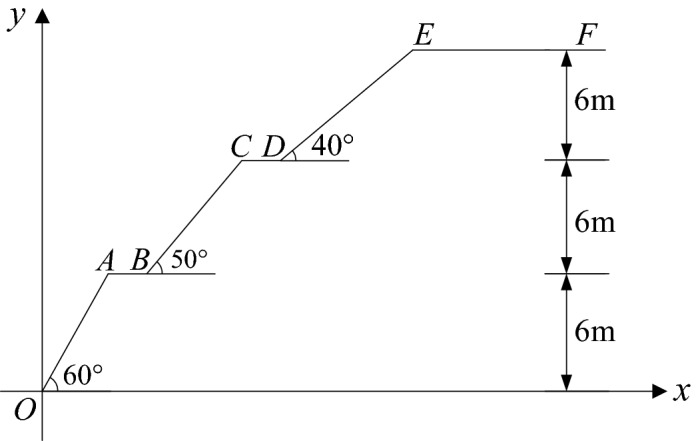
Dimension of the slope in the calculation example (unit: m).

We selected a multi-level loess slope described in the literature for comparative analysis^35,36^. The total height of the slope was 18 m, divided into three levels. Each level was 6 m tall, the platform width was 2 m, and the slope angles of the lowest, middle, and top levels from bottom to top were 60°, 50°, and 40°. The specific calculation model is shown in Fig. 7, and the geotechnical material parameters are shown in Table 1.

**Table 1:** Material parameters of calculation examples.

*γ* (kN/m^3^)	*c* (kPa)	*φ* (°)
16.5	16	23

### Calculation results of the brachistochrone sliding surface

**Figure 8:**
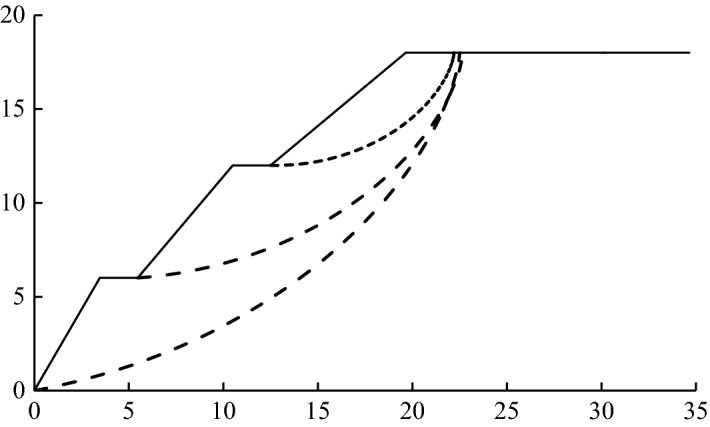
The position of the sliding surface of the steps at all levels (unit: m).

We carried out the sliding surface search and stability safety-factor calculation for the multi-level slope with a calculation program compiled by MATLAB. The potential sliding surface positions of the steps at each level are shown in Fig. 8, and the corresponding stability safety factors are shown in Table 2.

**Table 2:** Safety factors of the steps at all levels.

Step	Safety factor
First step	1.043
Second step	1.318
Third step	1.921

Looking at both Fig. 8 and Table 2, it becomes evident that the most dangerous sliding surface in this calculation example was the potential sliding surface corresponding to the first step. The sliding surface ran through the entire slope, the stability safety factor was 1.043, and the curve vertex coordinates were (22.5,18). The potential sliding surfaces corresponding to the second and third step were secondary sliding surfaces, the safety factors were 1.318 and 1.921 respectively, and the coordinates of the curve vertices were (22.7,18) and (22.2,18) respectively.

### Comparative analysis of the improved brachistochrone sliding surface

From the calculation results given in section "Calculation results of the brachistochrone sliding surface", it can be seen that the most dangerous sliding surface was the main sliding surface corresponding to the first step, and the coordinate of the sliding-in point was (22.5,18). To continue the search process, only the main sliding surface needed to be determined. In order to reduce the search workload, it was best to determine the search range of the sliding-in point near the coordinate point (22.5,18). Taking the abscissa of the sliding point as the central point, we searched for the sliding point to the left and right at intervals of 0.1 M. When the safety factor was smallest, the coordinates of the sliding point and the value of the improvement coefficient were the required results. During the searching calculation, when the curve-vertex coordinates of the potential sliding surface were located at (21.8,18) and the improvement coefficient *μ* was about 1.16, the stability safety factor was the minimum, 1.039.

**Figure 9:**
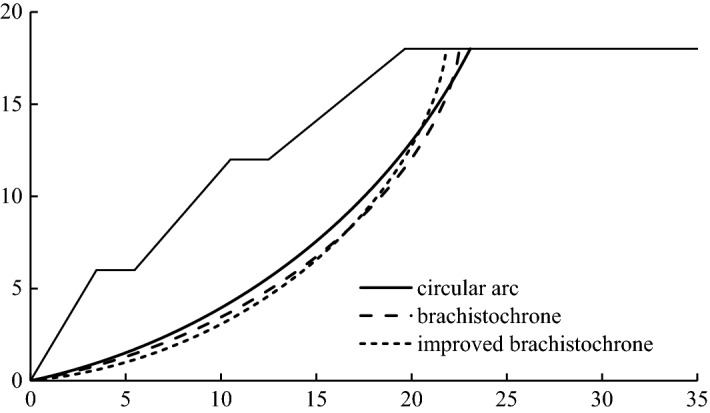
Comparison of sliding surface position as determined by different methods (unit: m).

**Figure 10:**
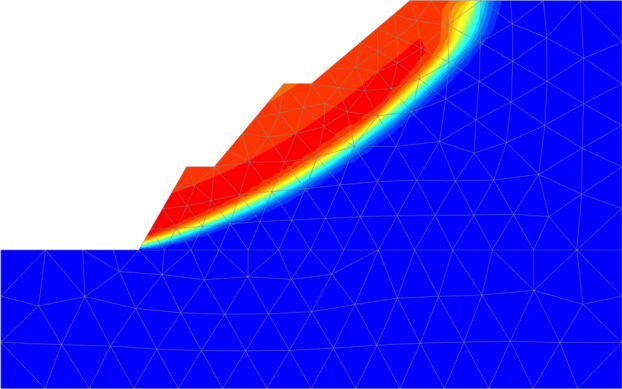
Incremental displacement nephogram of model.

At the same time, in order to fully verify the rationality of the brachistochrone sliding surface and the applicability of the brachistochrone sliding surface in practical engineering, we also used finite element software to conduct numerical simulation for the calculation example. The method used in the numerical simulation was the strength-reduction method. We then compared the sliding surface positions and stability safety factors obtained by different methods. The positional relationship is shown in Fig. 9, the numerical simulation calculation results are shown in Fig. 10, and the corresponding safety factors are shown in Table 3. Overall, the position of the sliding surface obtained using the brachistochrone and the improved brachistochrone was not only very close to the position of the classical circular arc sliding surface, but also very consistent with the slope deformation obtained by numerical simulation. The safety factors corresponding to these sliding surfaces were also very similar. This indicated that the brachistochrone sliding surface could be successfully applied in practical engineering.

**Table 3:** Comparison of safety factors obtained by different methods.

Slip surface form	References	Safety factor
Arc	Li Zhong^8^	1.18
	Janbu method	1.064
–	Finite element strength reduction method	0.996
Brachistochrone	This article (simplified Janbu method)	1.043
Improved brachistochrone	This article (simplified Janbu method)	1.039

Further, one can see from Fig. 9 that the sliding-in point of the brachistochrone sliding surface was (22.5,18), which was slightly closer to the airside compared with the sliding-in point of the circular arc sliding surface (23.1,18). After introduction of the improved coefficient, the sliding-in point of the improved brachistochrone sliding surface moved to (21.8,18) compared with the brachistochrone sliding surface. In terms of the curve shape, the circular arc sliding surface was relatively gentle, and the slope of the tangent line at the sliding-in point was obviously smaller than that of the sliding surface of the brachistochrone or the improved brachistochrone. The latter two sliding surfaces were relatively steep at the entry section at the back edge of the slope, and the slope of the overall slide arc changed greatly, which is basically below the classical circular arc slide surface. This characteristic was especially obvious for the brachistochrone after improvement; which was consistent with the characteristics of a loess slope with a steep rupture wall and a relatively steep slide surface. At the same time, comparing Figs. 9 and 10, one can see that the analysis results of the arc-shaped sliding surface and the brachistochrone sliding surface are almost consistent with the slope deformation obtained by numerical simulation.

Table 3 shows that the safety factor determined by the brachistochrone and the improved brachistochrone fell between the safety factors obtained by the circular arc sliding surface and the strength reduction methods, and the safety factor decreased after introduction of the improved factor. This indicated that the potential sliding surface searched by the improved brachistochrone was more ideal than the classic brachistochrone and the corresponding safety factor was smaller.

## Conclusions

In this study, we established a multi-level loess slope sliding surface search model for brachistochrone sliding surfaces. The sliding surface curve equation was improved and the potential sliding surface continuously searched for, and then the rationality of the approach was verified with a calculation example. The main conclusions are as follows:

1. Compared with the existing commonly used sliding surface curve form, the brachistochrone has a specific physical meaning.
2. Using the brachistochrone to search for the potential sliding surface can prevent the problem of finding the rotation center of a circular arc or logarithmic spiral sliding surface in the search process. The search only requires moving the positions of the two ends of the sliding surface, a simpler process which is easier to program for calculation and involves a small workload.
3. The established search model for the brachistochrone sliding surface of multi-level loess slopes can accurately and quickly search the potential sliding surface corresponding to each step in a multi-level slope. The position of the original brachistochrone sliding surface obtained and the improved brachistochrone sliding surface are in agreement with that of the classical arc. The stability safety factor is also similar, and the potential sliding surface after introduction of the improved coefficient is more ideal.
4. The results of this study provide a new form of sliding surface curve for slope-stability analysis, one which can significantly improve the efficiency of searches for potential slip surfaces in multi-level loess slopes.

## Data Availability

The datasets used and analysed during the current study available from the corresponding author on reasonable request.
